# ‘But what if you miss something …?’: factors that influence medical student consideration of cost in decision making

**DOI:** 10.1186/s12909-023-04349-3

**Published:** 2023-06-14

**Authors:** Emmanuel Tan, Wei Ming Ng, Poh Choong Soh, Daniel Tan, Jennifer Cleland

**Affiliations:** 1 Lee Kong Chian School of Medicine, Nanyang Technological University, Singapore, Singapore; 2 Department of Emergency Medicine, Ng Teng Fong General Hospital, Singapore, Singapore

**Keywords:** Cost, Cost-conscious care, Decision making, Medical students, Emergency department

## Abstract

**Context:**

Cost-conscious care is critical for healthcare sustainability but evidence suggests that most doctors do not consider cost in their clinical decision making. A critical step in changing this is understanding the barriers to encouraging behaviours and attitudes related to cost-conscious care. We therefore conducted a qualitative study to address the research question: what factors influence consideration of cost in emergency medicine (ED) clinical decision making?

**Methods:**

This was a qualitative focus group study using patient vignettes to explore attitudes towards cost-conscious clinical decision making. Participants were Year 4 and Year 5 medical students from Singapore, a country with a fee-for-service healthcare system. After a data-driven initial data analysis, and to make sense of a multitude of factors impacting on cost conscious care, we selected Fishbein’s integrative model of behavioural prediction to underpin secondary data analysis.

**Results:**

Via four focus groups with 21 participants, we identified five main themes relevant to the integrative model of behavioural prediction. These were: attitudes towards considering cost when managing a patient (e.g., “better safe than sorry”); normative beliefs (e.g., doing what others do, perceptions of patient wishes); efficacy beliefs (e.g., no authority to take decisions or challenge); skills and knowledge (e.g., little knowledge of costs), and environmental constraints (e.g., the nature of the healthcare system).

**Discussion:**

Medical students do not consider cost in their clinical decision making due to numerous factors, of which lack of knowledge of costs is but one. While some of the factors identified reflect those found in previous studies with residents and fully-trained staff, and in other contexts, theory driven analysis added value in that it facilitated a richer exploration of why students do not consider cost in clinical decision making. Our findings provide insight to inform how best to engage and empower educators and learners in teaching and learning about cost-conscious care.

**Supplementary Information:**

The online version contains supplementary material available at 10.1186/s12909-023-04349-3.

## Background

“There is a limit to the resources any society can devote to medical care” [1]. Although written more than 35 years ago, this statement is even more pertinent today when the relentless increase in health care costs is a threat to the future of health care in many different countries [2–4]. Yet, although cost-conscious care is clearly critical for healthcare sustainability, evidence suggests that most doctors do not consider the costs of tests, equipment, medications and so on in their clinical decision making [5–7].

This suggests a critical need for education and training on cost conscious care. Organisations such as the American Association of Medical Colleges (AAMC) have advocated for new residents to “incorporate cost awareness and principles of cost-effectiveness” in their diagnostic evaluations ([8] p19). This growing recognition of the importance of raising awareness of the cost of healthcare and effective use of limited healthcare resources is also apparent in other countries and contexts [9–12]. However, there is little guidance on when, where or how to incorporate teaching on cost conscious care. What studies do exist on this topic tend to focus on attitudes [13] and perceptions [14] towards costs conscious care, importance of health economics [15], and how this is taught [11, 16] rather than focusing specifically on considering cost and value in healthcare decision making.

A critical first step in changing behaviour and informing the content of teaching and learning materials to support change is understanding the factors which limit consideration of cost-conscious care. This might be determined by a variety of factors, including, for example, macro-level barriers such as the fear of malpractice lawsuits [17] and reimbursement system [18], or, at a more individual and group level, lack of clinical role models and the culture of the care team [19].

Given the relative paucity of prior research, our aim was to identify and explore factors influencing engagement in cost-conscious care in senior medical students. Our specific research question was: what are factors influencing consideration of cost in clinical decision making?

## Methods

### Design

We used a qualitative approach for data collection [20], specifically focus groups. Focus groups can generate data regarding perceptions and beliefs. Participants act to both challenge and support each other’s ideas [21] and the group setting can make people more confident in sharing information [22].

### Context

**Fig. 1:**
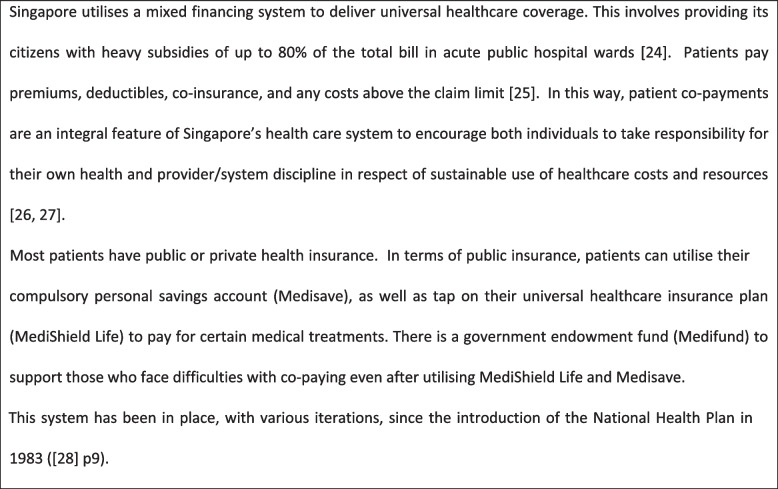
Brief overview of healthcare financing in Singapore [24–28]

The study setting was one of Singapore’s two undergraduate medical schools, Lee Kong Chian School of Medicine (LKCMedicine). At the time of carrying out this study LKCMedicine students were not formally taught about cost considerations or the economics of healthcare [23]. However, they were exposed to conversations about cost and healthcare from Year 3 onwards on clinical rotations in hospitals, clinics and general practices within Singapore’s fee-for-service public health service (See Fig. 1). Patient co-payment is an integral feature of this system. On admission, hospitals are required to provide patients with an estimate of their bill and patients must consider whether to progress with a recommended treatment once fully aware of the cost. Thus, seeing how cost is considered within clinical and patient decision making is part of the informal curriculum [23].

### Participants

We purposely recruited students who had completed at least one full year of clinical rotations and had completed, or were about to start, their Emergency Department (ED) rotation. We focused on the ED because EDs are often targeted for cost reductions [29–31] and it is not uncommon for ED patients to refuse emergency medical treatment on the grounds of cost in Singapore [32] and other contexts [33, 34].

After receiving ethical approvals, we advertised the study via year group invitation emails to Years 4 and 5 students. We followed up indications of interest with an email or WhatsApp, providing more study information as well as details of focus group time and place. As is standard practice locally, we gave participants a $10 gift voucher.

### Data collection

**Fig. 2:**
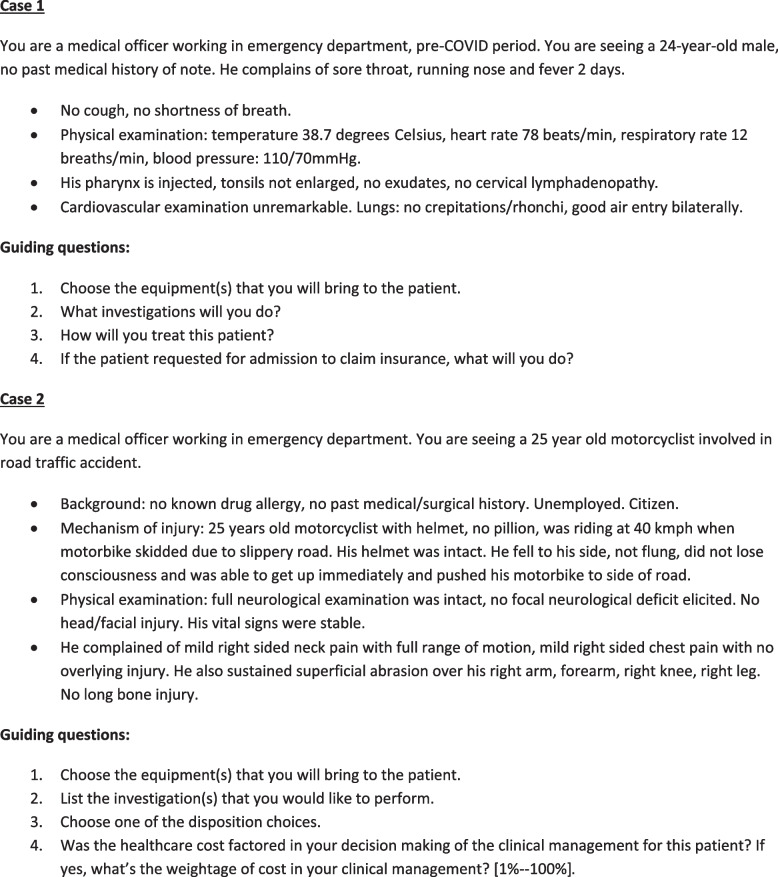
Two clinical vignettes reflecting “typical” ED encounters

Preliminary study discussions with clinical colleagues and knowledge of the literature suggested to us that students may not consciously consider cost in their clinical decision making. Thus, we designed two context-appropriate clinical vignettes plus guiding questions to orient participants to the issue(s) under study and to provide a focus for discussion [35]. These were structured to reflect two typical ED encounters (see Fig. 2). These were formulating diagnosis, selecting what equipment to use, and what, if any, investigations to order (see Additional file 1 for the full vignettes). We highlighted to participants that they were no absolute right or wrong answers: our focus was on understanding decision making rather than forcing participants to make a particular decision. The vignettes were piloted for appropriateness and “understandability” with a recent graduate working in ED.

We organised the focus group discussion into two parts. After introducing the study and reinforcing that participation was voluntary, we introduced the vignettes and encouraged discussion about best course of action. No cost information was provided. We then revealed the costs of equipment and laboratory test costs relevant to the vignettes, and asked participants if this new information would influence their clinical decision making. The researchers provided minimal prompting during discussions (participants fully engaged in the discussion based on the initial guiding questions). By the end of the third focus group, we felt that we had already gathered sufficient data to inform our research question. We undertook one further focus group and this confirmed little new information (participants’ comments were largely similar to those of earlier sessions). The focus groups were carried out between November 2021 and January 2022.

### Data management and analysis

Focus groups were audio-recorded with participants’ permission, anonymised through the transcription process, then entered into NVIVO Release 1.6.1 (QRS International Pty Ltd, Doncaster, Vic, Australia) qualitative data analysis software programme to facilitate multi-analyst data coding. ET conducted an initial, inductive, data-driven thematic analysis to identify meaningful subjects answering the research question, and condense the content of the data to key themes. Analysis progressed as collaboratively as a team, via regular team meetings by Zoom and email conversations where ongoing coding and comparisons were explored. The themes identified at this stage of the process are available upon reasonable request from the corresponding author.

**Fig. 3:**
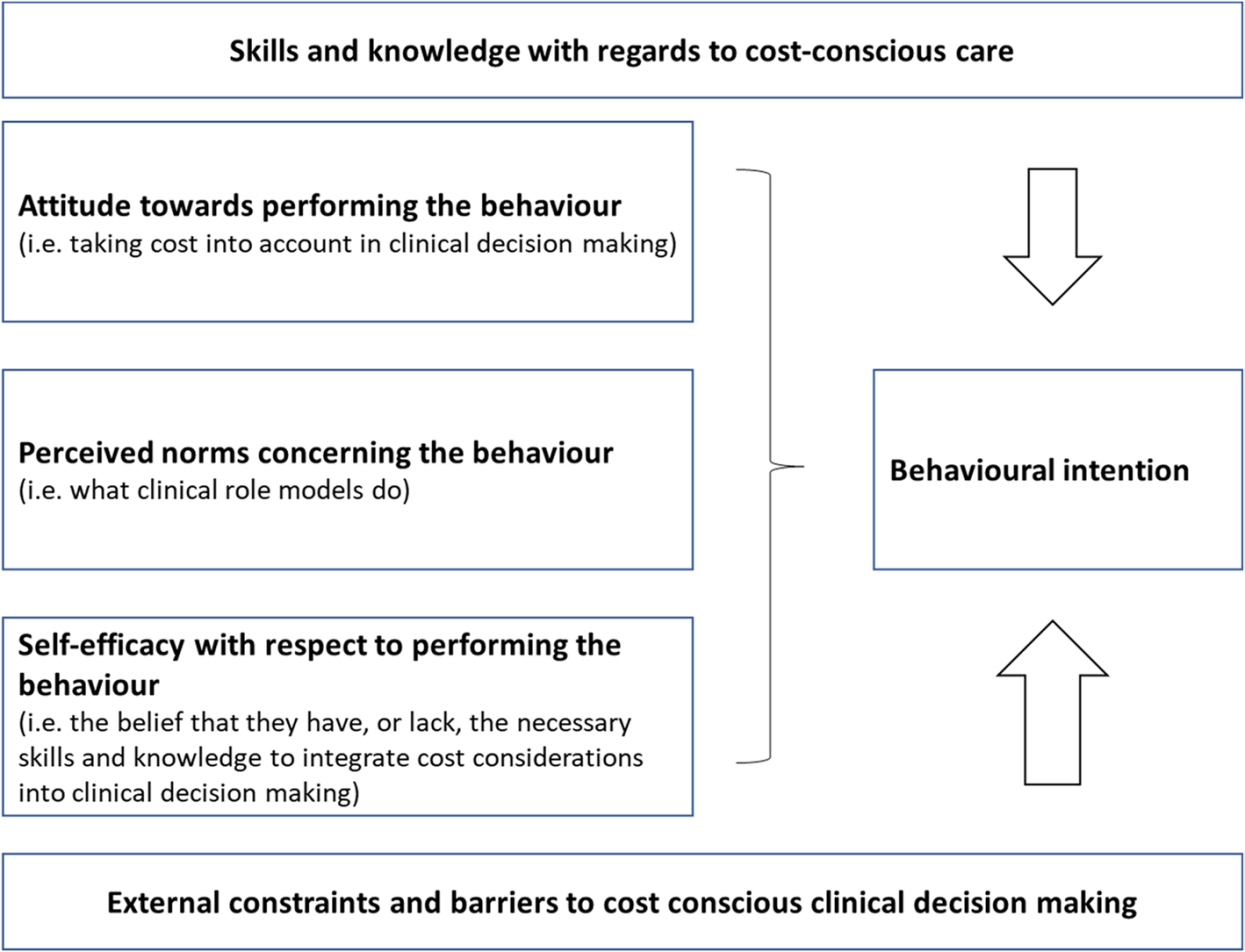
Primary determinants of behavioural intention to cost conscious decision making and care. Adapted from Fishbein’s integrative model of behavioural prediction [36]

During the process of thematic analysis, we were struck by the number of personal, social and environmental barriers to cost-conscious care raised by our participants. Given this, we then carried out a secondary, deductive data analysis using Fishbein’s integrative model of behavioural prediction as a framework [36]. This model proposes that there are three primary determinants of behavioural intention (Fig. 3): the attitude towards performing the behaviour, in this case taking cost into account in clinical decision making (e.g., the belief that doing so will lead to unfavourable outcomes for the patient or the student); perceived norms concerning the behaviour (e.g., what do others, particularly role models, do); self-efficacy with respect to performing the behaviour (e.g., the belief that they have, or lack, the necessary skills and knowledge to integrate cost considerations into clinical decision making). The more a student perceives that s/he has the necessary skills and knowledge to consider cost in clinical decision making, even in the face of constraints (e.g., lack of time), the stronger will be his/her intention to perform the behaviour. The model also considers the indirect role on influencing behaviour played by individual difference factors (e.g., personality). The integrative model of behavioural prediction has been used extensively to identify influences on healthcare professional and healthcare educator behaviours (e.g., [37–39]).

### Reflexivity

In qualitative research, it is important to consider the background of researchers and how this may influence data collection and analysis [40]. Our team included experts from medical education and emergency medicine. JC has a research interest in cost and value in medical education and has published extensively on this topic. NWM and SPC are clinicians with an active interest in ensuring clinically and cost-effective care in the ED. DT is a graduate of the medical school where this study is conducted and is hence familiar with the curriculum. ET holds a Master of Professional Accounting and worked in leading financial institutions before joining academia. The team regularly considered their positions and assumptions during collection and analysis [41].

### Ethical approval

The study protocol (IRB-2020–12-002) was approved by the Institutional Review Board of Nanyang Technological University.

### Patient and public involvement

The development of this research question and data collection was not directly informed by questioning of patients’ priorities, experience and preferences. Patients and the public were not involved in the study.

## Results

Twenty-one students participated in four focus group discussions. Each focus group lasted approximately 60 min. Participants were representative of their year groups in respect of gender and age (participant demographics can be found in Additional file 2).

Five main themes relevant to the integrative model of behavioural prediction [36] were identified from the focus group data. These were: attitudes towards incorporating cost into clinical decision making; normative beliefs and motivation to comply; self-efficacy beliefs; skills and knowledge, and environmental constraints.

### Attitudes and beliefs towards incorporating cost into clinical decision making

This theme refers to beliefs which link incorporating cost into clinical decision making to expected outcomes and the degree to which this is positively or negatively valued by the student. This includes the consequences of considering cost for both the student and the patient. It encompasses the student’s personal emotions and their belief in terms of the general value of considering cost, as well as their attitudes towards the usefulness of specific items which come with a cost.

Participants focused overwhelmingly on negative expected outcomes. They considered, for example, that not doing a particular investigation was an unnecessary risk that might lead to negative outcomes for the patient. They expressed their primary focus as the best interests of the patient and their job to rule out life-threatening causes of illness. Their overwhelming concern was patient safety and outcomes.

*I think I mean it’s a bit expensive, yeah, but if you miss an injury then it can be life threatening or like mobility threatening when it’s quite, uh, you know, quite unreasonable also. (P7)*

*I think it’s like, kind of like, better to be safe than sorry kind of mentality, for me. Yeah. I would rather do it and make sure the patient is okay than not do it and then like, um, if in the unfortunate event that the patient deteriorates. (P6)*

While participants were able to discuss cost and sustainability this seemed more on an abstract level: it was not part of their clinical decision-making process and instead was an administrative matter.

*We’re also quite detached from the cost, like we are not the ones handling the transactions. (P8)*

*Um, but I think in a public setting, I think our main, I think our main goal is just to work the patient up and see what we can do best for them. (P15)*

Rather their attitude was one of “better safe than sorry” (P6).

### Normative beliefs

Normative beliefs refer to the pressure to avoid not doing something (e.g., ordering a test) in terms of the student’s perceptions of what others think they should do, as well as the student’s perceptions of what other students are doing (see later). The most common reference group was that of consultants and other medical staff. Participants talked about following what they see others do, rather than making a cost–benefit evaluation. For example, if others typically order a certain test, so too will the students. For example,

*Can I just ask, why are we not setting a plug? Cause at the ED they set up plug for everyone. (P14)*

*Every patient comes in and gets the FBC and renal panel. I didn’t know why. (P1)*

Students also highlighted the role of patients in shaping their attitudes towards costs. They felt they should inform patients about the costs and let them decide whether or not to go ahead with treatment or investigations.

*I think we need to meet his expectation first like. He could well be like coming because he has some concerns and worries and he might expect investigation of us. (P18)*

Their perception of an individual patient’s financial situation, such as whether they could afford treatment or had sufficient insurance coverage, was discussed (e.g., *I think it will only matter to me if there’s like very extraordinarily expensive things or the patient needs to consistently follow up with like very expensive scans and all that. Or if the patient has financial difficulties* (P16)) but seemed to come secondary to a perception that if patients presented at the ED, these patients probably wished to receive treatment and investigations.

*He’s already at the emergency department and he’s probably worried about um, I mean he probably wants to get some answers and some treatment. So uh, would it be better to do some investigations to uh, like, uh, like put his mind at ease and then like, direct the management or so. (P6)*

### Efficacy beliefs (self-efficacy)

Self-efficacy refers to the belief, or confidence, that one can carry out a behaviour (in this case, cost conscious care) even under difficult circumstances. Low self-efficacy is related to doubting one’s own judgement or ability to carry out cost conscious care (or that one has the skills to do so [see Skills and knowledge]). Issues related to self-efficacy included participants feeling they had little authority in the ED and needed to clear their decisions with seniors.

*Number one, you’ll probably get screamed at by a consultant. Number two, the family will be super pissed at you, and number three, to top it off you probably won’t be able to forgive yourself or you’ll be very down after that, because you sort of like, you contributed to that. On the other hand, if you do it like pre-emptively in a sense, it’s not the most efficient use of resources, yes, unfortunately, but at the end of the day, you know, your salary is fixed and it’s okay, you’re covering your own ass. (P5)*

Self-efficacy is linked to attribution, and students did not want to be the cause of adverse outcomes. They had a strong tendency to over-investigate for fear of missing something that could cause harm to the patient (see also Beliefs and Attitudes), rather than basing their decisions on clinical probability. Participants discussed how they would order tests even where they were not fully certain such tests were needed to minimise the change of adverse clinical outcomes (e.g., *I would want to cover all my bases, just in case anything happens* (P19). They assumed that ED patients were serious cases, and this appeared to contribute to their arguably overly cautious approach.

### Skills and knowledge

This theme refers to participants feeling they have the necessary skills and knowledge to take cost into account in their clinical decision making. In terms of knowledge, they had very limited awareness of the cost of ED equipment (e.g., intravenous cannula, blood tube, syringes) or common laboratory and radiological investigations (e.g., liver function tests, chest X-ray, CT [Computed Tomography] scans).

*It is probably very expensive, I don’t know how expensive. (P3)*

When asked how much they thought these might be, they either could not estimate cost or their estimates were very inaccurate, usually much less than the actual cost.

*It sounds a bit stupid but I thought that most blood tests would be like around the same price but I see there’s like a big difference between like c-reactive protein vs procal, even though they kind of test for similar things. (P21)*

When accurate costs were shared in the second part of the vignette discussion, participants were very surprised. Participants reported that they were not taught the prices of equipment or investigations in the classroom or in the workplace.

*e.g., I feel like, uh, we’ve never really been, uh, educated on the prices of the tubes itself. And like, frankly speaking, it’s, it’s actually more expensive than what I expected it to be. (P11)*

In terms of skills, participants reported that their focus was on learning to successfully use equipment (e.g., successfully inserting an intravenous (IV) cannula into a patient’s vein for medication or fluids administration), rather than thinking beyond skills development to consider if the task was necessary.

### Environmental constraints

Environmental constraints refer to barriers to cost conscious clinical decision making or cost-conscious care. The themes were mostly associated with students being aware that they are training in a public hospital setting which has constrained resources (e.g., limited bed spaces). However, they argued that, since patients pay a standard price in a public hospital ED setting, they didn’t need to pay attention to the costs of the equipment or tests because these items are already included in the ED bill (e.g., *And I think since bedside ultrasound is also included in the ED bill already, we will just do it.* (P18)). They indicated that they might approach costs differently if they were practicing in a private hospital.

## Discussion

### Main findings

We identified a range of factors related to why medical students training in a fee-for-service public healthcare system may not consider cost in their clinical decision making. Lack of knowledge as to the cost of common medical investigations and equipment plays a role, but this is only part of the picture. Students are very focused on patient safety and outcomes and have the attitude that more is better (in order not to miss anything, no matter how unlikely). They believe that patients who come to the ED probably want treatment and thus they are behaving professionally by carrying out numerous tests and investigations. Even when provided with specific knowledge about equipment and investigation costs, participants still preferred to “do everything, look for everything” instead of incorporating cost into their decision making.

This attitude of erring on the side of (clinical) caution was strongly shaped by anxieties about, first, potential negative clinical outcomes if things were to go wrong and, second, getting the blame if it did. Students follow the norms of what they see and see themselves as powerless to make decisions anyway. Finally, students do not see their role as to be concerned with costs, seeing this as more of an administrative concern rather than a clinical one. The only time when they seemed to consider cost was at a patient-level (e.g., when they thought the patient could not afford an investigation or treatment), but there was little consideration of cost at a systems level.

### Comparison with previous literature

Our participants’ anxieties and perception of their roles as mere medical students powerless to make key clinical decisions are largely aligned to the literature on professional identity [42]. Reflecting one of the few previous studies in this area with medical students, albeit in the US context, our participants acknowledged the that physicians have a duty to contain costs yet their own clinical decisions are influenced by the fear of potential malpractice risks and what they see others do [43]. Some of the barriers identified reflect those found in previous studies with residents [44, 45] and fully-trained staff [46, 47], and in other contexts [48, 49]. Nevertheless, our theory driven analysis added value in that it facilitated a richer exploration of why students do not consider cost in clinical decision making.

### Implications for research, policy and practice

Although qualitative research does not stake a claim to generalisability in the way quantitative research does, our findings have educational and research implications. A gap in knowledge about healthcare costs is clearly not being addressed by workplace learning and role modelling. This suggests the need for formal teaching and learning about cost-conscious care. Incorporating this into the formal curriculum will send a message that cost consideration is important within medical practice. However, it is also critical to align messages across the formal, informal and hidden curricula, with the last of these potentially the most influential given it is based on values, attitudes and norms [23] – all factors identified in our study as important to considering (or not) cost in clinical decision making.

On one hand, we could argue that our participants were acting in their patients’ best interests by leaving no stone unturned. On the other hand, it is not good clinical practice to over-investigate or to not use a balance of probability – and by not doing so, incurring more charges for the patient and the system. If we want the doctors of tomorrow to engage in cost-conscious care we have to support them not only in knowing costs (knowledge), but also in learning how to balance cost and value to the patient, and accepting that cost-conscious care is everyone’s concern. One way to do this might be working with students to explore potentially conflicting dilemmas around care and cost, and how to manage these, might be a fruitful method of achieving behaviour change (e.g., [50]).

Our findings suggest a level of unconscious incompetence [51]. Participants did not recognise or consider it an issue that they were cost-naïve in their clinical decision making. Individuals must recognize their own incompetence, and the value of the new skill, before moving on to the next stage. But there must be a stimulus to change. If students and junior doctors do not see their role models (more senior clinicians) including cost into clinical decision making, they will not have the incentive to do so themselves. It may be that interventions to encourage cost-conscious care are needed for those teaching medical students and junior doctors in the workplace as well as the learners themselves.

### Strengths and limitations of this study

Our study is set in the ED and our findings may not be transferable to other medical departments. It is also set in one country with a particular healthcare financing model. Our study involved medical students not fully trained doctors for whom the influencing factors and barriers may be different. These points may limit our study’s generalisability to other contexts. However, the purpose of qualitative research is not statistical generalisation and our focus, cost-conscious care, is not unique to the context of this study. Moreover, our use of a theory of behaviour change to interpret the data facilitated a richer exploration of students’ failure to consider cost in their behaviour and aids conceptual generalisability [52]. Our use of theory also adds more generally to the literature on cost-conscious care, which, to date, has tended to be descriptive or quantitative [53, 54]. Our relatively small qualitative study was not able to address how culture, gender, personality and other individual factors may lead to different attitudes towards cost conscious care. However, even with a relatively small number of participants, we have high information power [55], because of a narrow study aim, a specific sample, good quality data and theory-driven analysis. Of course, as with any voluntary study, there would have been an element of participant self-selection and we have no idea as to whether our participants were particularly cost- naive or cost conscious.

## Conclusion

This study contributes to a wider conversation in the literature about cost and value medical education. Our findings provide insight to inform how best to engage and empower educators and learners in teaching and learning about cost-conscious care. Embracing cost and value education has the potential to open up new areas of scholarship and ensure that as a professional group we are able to contribute to improving the value of healthcare education and delivery.

## Supplementary Information

**Additional file 1.** Full clinical vignettes.

**Additional file 2.** Participant demographics.

## Data Availability

The datasets are available upon reasonable request by contacting the corresponding author.
