# Supplementary material for: ‘But what if you miss something …?’: factors that influence medical student consideration of cost in decision making
Source: BMC Med Educ. 2023 Jun 14;23:437. doi: 10.1186/s12909-023-04349-3 (PMC10268427; doi:10.1186/s12909-023-04349-3)
Supplement: Supplementary file 1 — Additional file 1. Full clinical vignettes. [file 12909_2023_4349_MOESM1_ESM.docx]

**Additional file 1: Full clinical vignettes**

**Case 1**

***Background***

You are a medical officer working in emergency department, pre-COVID period. You are seeing a 24-year-old male, no past medical history of note. He complains of sore throat, running nose and fever 2 days.

- No cough, no shortness of breath.
- Physical examination: temperature 38.7 degrees Celsius, heart rate 78 beats/min, respiratory rate 12 breaths/min, blood pressure: 110/70mmHg
- His pharynx is injected, tonsils not enlarged, no exudates, no cervical lymphadenopathy
- Cardiovascular examination unremarkable. Lungs: no crepitations/rhonchi, good air entry bilaterally.

***Question 1: Please choose the equipment(s) that you will bring to the patient.***

- Intravenous cannula (blue – 22G)
- Intravenous cannula (pink – 20G)
- Intravenous cannula (green – 18G)
- Needle (blue – 22G)
- Needle (green – 18G)
- 3 mls syringe
- 5 mls syringe
- 10 mls syringe
- Butterfly venopuncture set
- Alcohol swabs
- Tegaderm
- Intravenous cannula stopper
- Torniquet
- Purple top blood tube (full blood count (FBC): 1 mls)
- Yellow top blood tube (renal panel, liver function test (LFT), procalcitonin (procal), c-reactive protein (CRP): 2 mls)
- Blue top blood tube (coagulation panel: 3 mls)
- Pink top blood tube (crossmatch: 1 mls)
- Blood gas tube

***Question 2: What investigations will you do?***

a. Labs:

- Full blood count (FBC)
- Renal Panel
- Liver Function
- NIL

b. Radiological:

- Chest X-ray (CXR)
- NIL

c. Others:

- Respiratory virus panel
- NIL

***Question 3: How will you treat this patient?***

a. Disposition:

- Treat and discharge
- Refer Specialist Outpatient Care (SOC)
- Admit 24 hrs ward (Extended ED Care ward)
- Admit inpatient

 b. Medications:

- Paracetamol
- Lozenges
- Mouth gargle
- Antibiotics
- Tamiflu
- Cough mixture
- NIL

***Question 4:*** ***If the patient requested for admission to claim insurance, what will you do?***

**Case 2**

***Background***

You are a medical officer working in emergency department. You are seeing a 25 year old motorcyclist involved in road traffic accident.

- Background: no known drug allergy, no past medical/surgical history. Unemployed. Citizen.
- Mechanism of injury: 25 years old motorcyclist with helmet, no pillion, was riding at 40 kmph when motorbike skidded due to slippery road. His helmet was intact. He fell to his side, not flung, did not lose consciousness and was able to get up immediately and pushed his motorbike to side of road.
- Physical examination: full neurological examination was intact, no focal neurological deficit elicited. No head/facial injury. His vital signs were stable.
- He complained of mild right sided neck pain with full range of motion, mild right sided chest pain with no overlying injury. He also sustained superficial abrasion over his right arm, forearm, right knee, right leg. No long bone injury.

***Question 1: Please choose the equipment(s) that you will bring to the patient.***

- Intravenous cannula (blue – 22G)
- Intravenous cannula (pink – 20G)
- Intravenous cannula (green – 18G)
- Needle (blue – 22G)
- Needle (green – 18G)
- 3 mls syringe
- 5 mls syringe
- 10 mls syringe
- Butterfly venopuncture set
- Alcohol swabs
- Tegaderm
- Intravenous cannula stopper
- Torniquet
- Purple top blood tube (full blood count (FBC): 1 mls)
- Yellow top blood tube (renal panel, liver function test (LFT), procalcitonin (procal), c-reactive protein (CRP): 2 mls)
- Blue top blood tube (coagulation panel: 3 mls)
- Pink top blood tube (crossmatch: 1 mls)
- Blood gas tube

***Question 2: Please list the investigation(s) that you would like to perform.***

- Full blood count
- Renal panel
- Liver function test
- Amylase
- Coagulation profile
- Group cross match
- Troponin I
- Blood culture set
- Arterial blood gas
- Venous blood gas
- Chest X-ray
- Pelvic X-ray
- Cervical spine X-ray
- Left oblique chest X-ray
- Right oblique chest X-ray
- Shoulder X-ray A/P + lateral (L/R)
- Humeral X-ray A/P + lateral (L/R)
- Elbow X-ray A/P + lateral (L/R)
- Radius/ulna X-ray A/P + lateral (L/R)
- Femur X-ray A/P + lateral (L/R)
- Knee X-ray A/P + lateral (L/R)
- Tibia/fibula X-ray A/P + lateral (L/R)
- CT brain
- CT cervical spine
- CT thorax
- CT abdomen
- CT pelvis
- Bedside ultrasound (inclusive in ED bills)

***Question 3: Please choose one of the disposition choices.***

- Discharge with no follow-up
- Discharge with follow up to polyclinic / general practitioners (GP)
- Admission to ED short stay ward
- Admission to inpatient ward

***Question 4: Was the healthcare cost factored in your decision making of the clinical management for this patient?* *If yes, what’s the weightage of cost in your clinical management? [1%--100%]***
