# Supplementary material for: ‘But what if you miss something …?’: factors that influence medical student consideration of cost in decision making
Source: BMC Med Educ. 2023 Jun 14;23:437. doi: 10.1186/s12909-023-04349-3 (PMC10268427; doi:10.1186/s12909-023-04349-3)
Supplement: Supplementary file 2 — Additional file 2. Participant demographics. [file 12909_2023_4349_MOESM2_ESM.docx]

**Additional file 2: Participant demographics**

| **Characteristic** |  |
| --- | --- |
| **Total number of participants** | 21 |
| **Mean age of participants** | 23 |
| **Participants per focus group** |  |
| Focus group 1 | 4 |
| Focus group 2 | 5 |
| Focus group 3 | 6 |
| Focus group 4 | 6 |
| **Gender** |  |
| Male | 14 |
| Female | 7 |
| **Year at medical school** |  |
| Year 4 | 18 |
| Year 5 | 3 |
